# Lipoprotein Lipase (LPL) is Associated with Neurite Pathology and Its Levels Are Markedly Reduced in the Dentate Gyrus of Alzheimer’s Disease Brains

**DOI:** 10.1369/0022155413505601

**Published:** 2013-12

**Authors:** Huilin Gong, Weijiang Dong, Steven W. Rostad, Santica M. Marcovina, John J. Albers, John D. Brunzell, Simona Vuletic

**Affiliations:** Northwest Lipid Metabolism and Diabetes Research Laboratories, Department of Medicine, School of Medicine, University of Washington, Seattle, WA (HG, WD, SMM, JJA, SV); CellNetix Pathology, Seattle, WA (SWR); Department of Medicine, School of Medicine, University of Washington, Seattle, WA (JDB)

**Keywords:** lipoprotein lipase, human brain, Alzheimer’s disease, neurons, glia, cerebrospinal fluid

## Abstract

Lipoprotein lipase (LPL) is involved in regulation of fatty acid metabolism, and facilitates cellular uptake of lipoproteins, lipids and lipid-soluble vitamins. We evaluated LPL distribution in healthy and Alzheimer’s disease (AD) brain tissue and its relative levels in cerebrospinal fluid. LPL immunostaining is widely present in different neuronal subgroups, microglia, astrocytes and oligodendroglia throughout cerebrum, cerebellum and spinal cord. LPL immunoreactivity is also present in leptomeninges, small blood vessels, choroid plexus and ependymal cells, Schwann cells associated with cranial nerves, and in anterior and posterior pituitary. *In vitro* studies have shown presence of secreted LPL in conditioned media of human cortical neuronal cell line (HCN2) and neuroblastoma cells (SK-N-SH), but not in media of cultured primary human astrocytes. LPL was present in cytoplasmic and nuclear fractions of neuronal cells and astrocytes *in vitro*. LPL immunoreactivity strongly associates with AD-related pathology, staining diffuse plaques, dystrophic and swollen neurites, possible Hirano bodies and activated glial cells. We observed no staining associated with neurofibrillary tangles or granulovacuolar degeneration. Granule cells of the dentate gyrus and the associated synaptic network showed significantly reduced staining in AD compared to control tissue. LPL was also reduced in AD CSF samples relative to those in controls.

## Introduction

Lipoprotein lipase (LPL) is secreted as a homodimer and acts as a rate-limiting enzyme that releases fatty acids from mono-, di- and triacylglycerides. It also promotes lipid exchange between apolipoprotein B (apoB) lipoproteins (low density lipoproteins, LDL; very low density lipoproteins, VLDL; chylomicrons) and between different high density lipoprotein (HDL) subclasses (Obunike et al. 1994; Goti et al. 2002; Mead et al. 2002; Shi et al. 2010). LPL also binds to the cell surface heparan sulfate proteoglycans, and thus forms a bridge between lipoprotein particles and cell surface receptors involved in lipoprotein particle uptake, which facilitates cellular uptake of lipoproteins, lipoprotein-associated lipids and lipophilic vitamins (Mead et al. 2002). These functions of LPL are well-defined in the periphery. However, published studies have shown that LPL and its co-factor, apoC-II, are also expressed in the brain of different mammalian species (Chajek et al. 1977; Brecher and Kuan 1979; Eckel and Robbins 1984; Semenkovich et al. 1989; Ben Zeev et al. 1990; Vilaro et al. 1990; Nunez et al. 1995; Blain et al. 2006), and the functions of LPL in the central nervous system (CNS) are still under investigation (Wang and Eckel 2012).

The CNS under normal conditions does not contain triacylglycerides (TAG) or apoB-containing lipoproteins (Adams and Bayliss 1968; Pitas et al. 1987; Agranoff et al. 1999; Dreissig et al. 2009). These differences between the brain and the periphery would, therefore, somewhat restrict the classic functions of LPL in the brain to those that are independent of TAG or apoB-containing lipoproteins. Brain lipoproteins consist of HDL-like particles, with apoE being the main apolipoprotein component (Pitas et al. 1987). Numerous studies have shown that apoE-containing lipoproteins bind to the so-called LDL receptors in the brain, leading to lipid uptake (Pitas et al. 1987; Lorent et al. 1995; Fagan et al. 1996). Binding to these receptors depends upon the nature of the protein components in the lipoproteins. Thus, the LDL and VLDL receptors in the brain bind HDL-like lipoproteins containing apoE (Pitas et al. 1987; Lorent et al. 1995; Fagan et al. 1996). In the periphery, the apolipoprotein-receptor interaction is facilitated by LPL (Eisenberg et al. 1992; Medh et al. 1996; Mead et al. 2002; Merkel et al. 2002), and this LPL function is likely to also remain relevant in the brain. LPL-facilitated uptake of lipoproteins is potentially of high importance in the brain, because lipoprotein uptake by neuronal cells is relevant for structural and functional integrity of synapses (Mauch et al. 2001; Göritz et al. 2005; Fester et al. 2009), and changes in lipoprotein metabolism have been shown to play a role in the pathogenesis of different brain diseases (Danik et al. 1999; Hayashi, 2011).

Furthermore, despite the virtual absence of triglycerides in human brain, diacylglycerides (DAG) play an important role in brain metabolism through their actions in regulating cell signaling pathways responsible for synaptic functions, cell differentiation, apoptosis and other cellular processes in the brain (Brose et al. 2004; Oudin et al. 2011). The LPL-dependent hydrolysis of diglycerides is likely an important mechanism for regulation of diglyceride availability in the brain. Therefore, it is important to understand the range of differences in LPL levels and function in human brain, particularly because LPL polymorphisms have been implicated in pathogenesis of stroke, Alzheimer’s disease, schizophrenia and other brain disorders (Myllykangas et al. 2001; Blain and Poirier 2004; Papassotiropoulos et al. 2005; Blain et al. 2006; Kostomarov et al. 2008; Wang C. et al. 2011; Xie et al. 2011; Munshi et al. 2012).

Previously published studies have detailed the distribution of LPL mRNA and protein in rodent brain tissue (Goldberg et al. 1989; Yacoub et al. 1990; Tavangar et al. 1992; Vilaro et al. 1990; Paradis et al. 2004a; Wang and Eckel 2012). In this study, we evaluated LPL distribution in human brain and in human brain cells (neurons and astrocytes) *in vitro*, and assessed differences in the distribution and levels of LPL in brain tissue and cerebrospinal fluid obtained from patients with Alzheimer’s disease (AD) and compared with control samples.

## Materials and Methods

### Materials

Vectastain ABC (avidin biotin complex) Elite kit (PK-6200), Antifade fluorescence mounting medium with 4,6-diamino-2-phenylindole (DAPI; H-1500), 3,5’-diaminobenzidine (DAB) staining kit (SK-4100), and biotinylated antibodies were purchased from Vector Laboratories Inc. (Burlingame, CA). Monoclonal antibody against LPL, clone 5D2 (Chang et al. 1998) was used in all immunohistochemical (IHC) and blotting experiments. Antibodies against beta tubulin (50-360-156), glial fibrillary acidic protein (GFAP; 01674441), CD68 (PIPA532330) and neuron-specific enolase (NSE; PIPA512373), xylenes (X4-4), Meyer’s hematoxylin (TA-125-MH), Bioworld antibody dilution buffer (50-199-075), Permount mounting medium (SP15-500) and SuperSignal West Femto kit (PI34095) were purchased from ThermoFisher Scientific (Houston, TX). Fluorescent secondary antibodies Alexa Fluor 488 (A21200) and 546 (A11035) were purchased from Life Technologies (Carlsbad, CA). An antibody against TATA-binding protein (TBP; A301-229A) was purchased from Bethyl Laboratories (Montogomery, TX). TrueBlot anti-mouse and anti-rabbit antibodies (29165 and 28926, respectively) were obtained from Rockland Immunochemicals (Gilbertsville, PA). Criterion XT polyacrylamide 4-12% gels (345-0123), XT MOPS running buffer (161-0788), XT reducing agent (161-0792) and extra-thick blotting paper (1703967) were purchased from Bio-Rad (Hercules, CA). An electric digital pressure cooker and antigen retrieval solution (EDTA) were obtained from Bio SB (Santa Barbara, CA).

### Brain Tissue and Cerebrospinal Fluid Samples

The study was approved by the University of Washington Human Subjects Division. Formalin-fixed, paraffin-embedded (FFPE) brain tissue slides were obtained from control subjects with no significant pathology upon neuropathological examination (*n*=20; 9 females, 11 males; age range, 61-101 years), and from neuropathologically verified Alzheimer’s disease subjects (*n*=20; 10 females, 10 males; age range, 63-97 years) from the University of Washington Neuropathology Core, Brain Aging and Neurodegeneration Brain Bank.

Cerebrospinal fluid (CSF) was obtained from the University of Washington Alzheimer Disease Research Center (ADRC) CSF bank. CSF was collected by lumbar puncture between the hours of 0900 and 1200 from control (*n*=27; 9 females, 18 males, age range, 61-86 years) and Alzheimer’s disease patients (*n*=27; 12 females, 15 males; age range, 65-87 years). Subjects fasted from midnight prior to CSF collection. Subjects were maintained in a supine position for l h before lumbar puncture. Subjects were then placed in the lateral decubitus position and the L3-4 or L4-5 interspace was infiltrated with 1% lidocaine to provide local anesthesia. CSF was collected with a 25-gauge Quincke spinal needle as 1 ml aliquots into polypropylene tubes, frozen immediately on dry ice and stored at -75C until needed for analyses. The samples were assessed for blood contamination (total protein, albumin, glucose, cell count, red blood cell count, apoB), and the analyses documented that the blood-brain barrier was not compromised in any of the tested subjects.

### Cell Culture

Primary human astrocyte precursors, human neuroblastoma cell line SK-N-SH (ATCC), and human cortical neurons, HCN2 (ATCC) were grown at 37C, 5% CO_2_, 95% humidity, in serum-supplemented media (DMEM). Primary human astrocyte precursors were incubated with G5 supplement (Gibco/Life Technologies, Cat. No. 17503-012) for 24 h prior to the experiment. For experimental purposes, cells were incubated for 24 h in serum-free media. Following incubation, conditioned media were collected, briefly centrifuged to pellet the cells, and frozen in aliquots for further analyses. Cells were washed with PBS and used for protein isolation.

### Protein Isolation

Proteins were isolated using NE-PER kit according to the manufacturer’s instructions, in the presence of HALT phosphatase and protease inhibitor cocktails (ThermoFisher Scientific, 78835 and 78430, respectively).

### Immunohistochemistry

Areas of study included all major brain regions – frontal, parietal, temporal and occipital cortices, anterior cingulate, hippocampus, amygdala, striatum, thalamus, midbrain, pons, medulla, cerebellum, spinal cord and pituitary. FFPE samples were deparaffinized and rehydrated in xylene and ethanol. Endogenous peroxidase activity was quenched using 3% H_2_O_2_ for 20 min for standard IHC protocol, followed by antigen retrieval by cooking the samples under pressure in EDTA solution (Bio SB, Santa Barbara, CA) for 8 min. Samples were cooled for 15 min, and tissue was incubated with anti-LPL antibody (7 µg/ml; 1:150) diluted in antibody dilution buffer for 2 h at room temperature in a humidified chamber, followed by 1 h incubation with biotinylated anti-mouse antibody (5 µg/ml; 1:200) and application of the ABC Elite kit (30 min; 1:200) according to the manufacturer’s instructions. The samples were stained using the DAB kit, counterstained with Mayer’s hematoxylin, dehydrated and coverslipped. The slides were observed using an Olympus microscope BX51 (Olympus Optical Co. Ltd, Tokyo, Japan), and micrographs taken using Kodak Mark EOS 5D camera and EOS utility software (Kodak, Rochester, NY). Densitometry analyses were performed using ImageJ software (http://rsb.info.nih.gov/ij/), and Image Pro Plus software (http://www.mediacy.com/index.aspx?page=IPP).

The fluorescent staining protocol omitted endogenous peroxidase quenching and the concentration of primary antibodies (anti-LPL, anti-GFAP, anti-NSE and anti-CD68) were increased (10 µg/ml; 1:100). No amplification of the signal was required, and slides were incubated with fluorescently labeled Alexa Fluor 488 (chicken anti-mouse) and 546 (goat anti-rabbit) antibodies for 30 min at room temperature (1:200). Slides were mounted using Anti-Fade mounting medium with DAPI, and micrographs taken using Nikon TiE microscope, Coolsnap ES^2^ camera and merged using NIS Elements software (Nikon, Tokyo, Japan).

### Western Blotting

CSF and brain tissue homogenates from control and AD subjects, and conditioned media, cytoplasmic and nuclear proteins from HCN2 cells, SK-N-SH and primary human astrocytes were resolved by SDS-PAGE on Criterion XT 4-12% gels, transferred to nitrocellulose membranes by semi-dry transfer (Bio-Rad), and incubated with anti-LPL antibody (1:1000) and antibodies for loading control (anti-β-tubulin for cytoplasmic and anti-TBP for nuclear proteins; 1:2000). The blot was developed using SuperSignal West Femto and documented by Kodak ImageStation CF400. Densitometry analyses were performed using ImageJ software (http://rsb.info.nih.gov/ij/), and data analyzed using t-test. P-values <0.05 were considered statistically significant.

## Results

**Figure 1.**
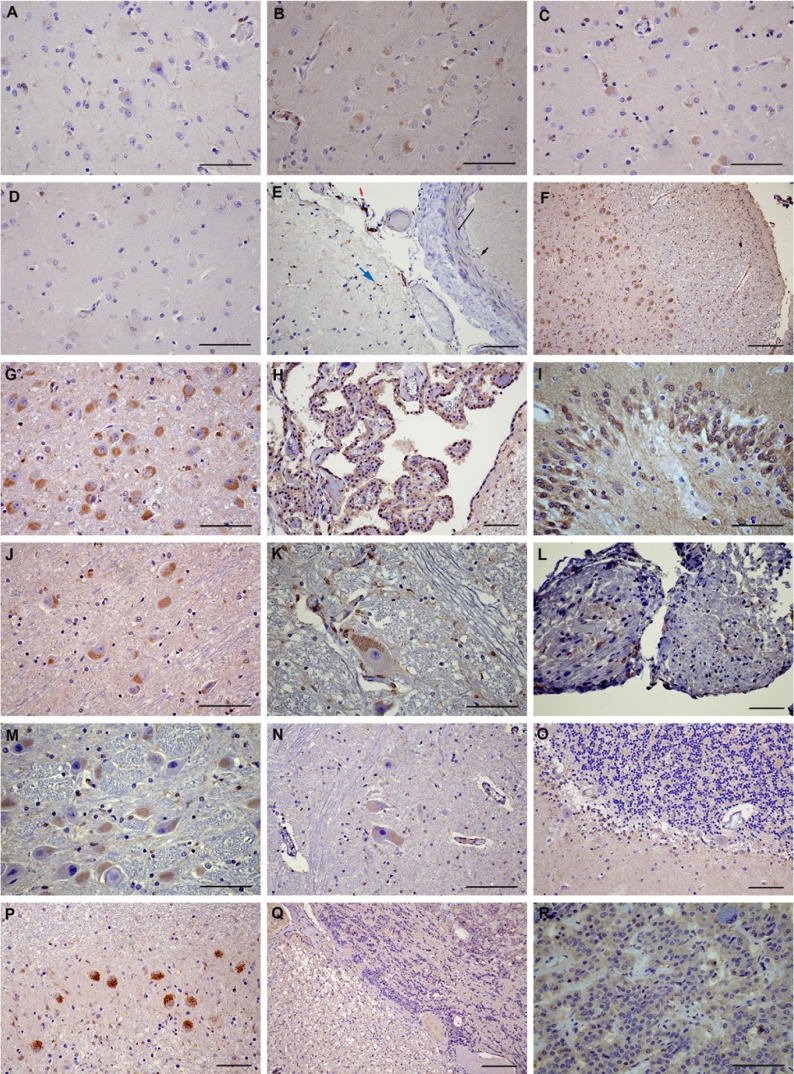
LPL in normal human brain. (A) Frontal cortex. (B) Parietal cortex. (C) Temporal cortex. (D) Occipital cortex. (E) Leptomeningae. Long black arrow, smooth muscle cells; short black arrow, endothelial cell; blue arrow, subpial microglia; red arrow, arachnoid. (F) Low magnification of hippocampus. (G) Dentate gyrus. (H) Hippocampal neurons. (I) Choroid plexus. (J) Thalamus. (K) Midbrain, third cranial nerve nucleus. (L) Cranial nerves with Schwann cells. (M) Medulla. (N) Spinal cord. (O) Cerebellum. (P) Cerebellum, dentate neurons. (Q) Pituitary, anterior (right) and posterior (left). (R) Anterior pituitary, detail. Scale bars = 50 µm (A-E, G-P and R) and 100 µm (E and Q).

We did not observe any marked differences in LPL immunoreactivity patterns or intensity related to age or sex in our samples. LPL was found in all major cortical areas (frontal, parietal, temporal and occipital lobes) (Fig. 1A-D). LPL immunoreactivity was present in leptomeninges, where it associated with smooth muscle cells, endothelial cells, some arachnoid cells and microglia/macrophages (Fig. 1E). Immunostaining was strongly associated with microglia in the subpial molecular layer. In the gray matter areas of the cortical regions, LPL was present in subgroups of neurons, mostly in the medium-size neurons of the upper-middle layer of the cortical areas, with diffuse cytoplasmic staining filling a large portion of the cell, with occasional nuclear staining. Among the major cortical regions, the occipital lobe had the lowest neuronal immunoreactivity for LPL, with scattered groups of positive neurons. Sporadic cytoplasmic and nuclear staining in astrocytes was observed throughout the cortical gray matter areas, as well as an extensive microglia cytoplasmic and nuclear LPL immunoreactivity in all examined gray matter areas. One of the dominant features of LPL immunoreactivity in the brain is a pervasive diffuse staining of the synaptic network, with finely delineated cellular processes outlining the underlying architecture of the tissue.

White matter areas throughout the brain featured extensive microglial staining, both cytoplasmic and nuclear, with more restrictive immunoreactivity in astrocytes and oligodendroglia. Small blood vessels in the brain tissue showed strong staining of the smooth muscle cells and endothelial cells.

In the hippocampus (Fig. 1F-H), LPL immunoreactivity was strongly positive in the ependymal cells, predominantly in the apical area. Extensive microglial staining with some positive astrocyte immunoreactivity was also characteristic in this region. The dentate gyrus displayed robust neuronal and the synaptic network staining (Fig. 1G), and neurons in the Cornu Ammonis (CA) layers 1, 2, 3 and 4 were also positive, with no significant differences in the intensity of staining between different CA neuronal groups. LPL immunoreactivity was found in the subicular neurons and microglia, with limited astrocyte staining. As seen in other brain areas, nuclear staining was present in both neuronal and glial cells. The parahippocampal cortex had a similar immunostaining pattern and the choroid plexus epithelium showed high LPL immunoreactivity (Fig. 1I).

Neurons in the lateral geniculate were very strongly positive for LPL, as well as a subset of neurons in the amygdala. LPL immunostaining was also strong in the striatal neurons, whereas thalamic neurons showed moderate to strong staining (Fig. 1J). In all of these regions, microglial staining was extensive, whereas astrocytic immunoreactivity was sporadic.

In the midbrain, presence of the pigment in neurons of substantia nigra prevented visualization of potential LPL staining. The red nucleus and the midline nuclei showed low to moderate neuronal staining. LPL immunoreactivity was also found in the third cranial nerve nucleus (Fig. 1K). The pattern of glial staining was similar to the other brain regions, with extensive microglial and scattered astrocyte staining.

The presence of the pigment in neurons in the locus coeruleus also prevented visualization of LPL immunostaining. Pontine nuclei showed positive LPL immunoreactivity, and some positive staining was visible in the Schwann cells associated with cranial nerves (Fig. 1L).

The medulla showed positive, albeit relatively weak, staining in the nuclei of the cranial nerves 9, 10 and 12. Strong LPL staining was present in the medium to large neurons in the medulla (Figure 1M), including the inferior olivary nuclei. The white matter staining pattern was similar to that observed in other brain regions, although the intensity of the white matter staining was stronger in the medulla than in other examined brain areas. Neurons in the spinal cord were weakly stained in the anterior horn, and some scattered neuronal staining in other areas could be observed (Fig. 1N).

Cerebellar dentate neurons were strongly immuno-positive for LPL, whereas granular neurons and molecular layer showed weakly positive staining (Fig. 1O-P). The internal granule cells showed positive nuclear staining but Purkinje cells showed weak LPL immunoreactivity. As in the other brain regions, there was an extensive diffuse staining along the cellular processes.

The pituitary gland was strongly positive for LPL in both the anterior and posterior pituitary (Fig. 1Q-R). Epithelial cells in the anterior pituitary were strongly stained, as well as neuronal processes and glia in the neurohypophysis.

**Figure 2.**
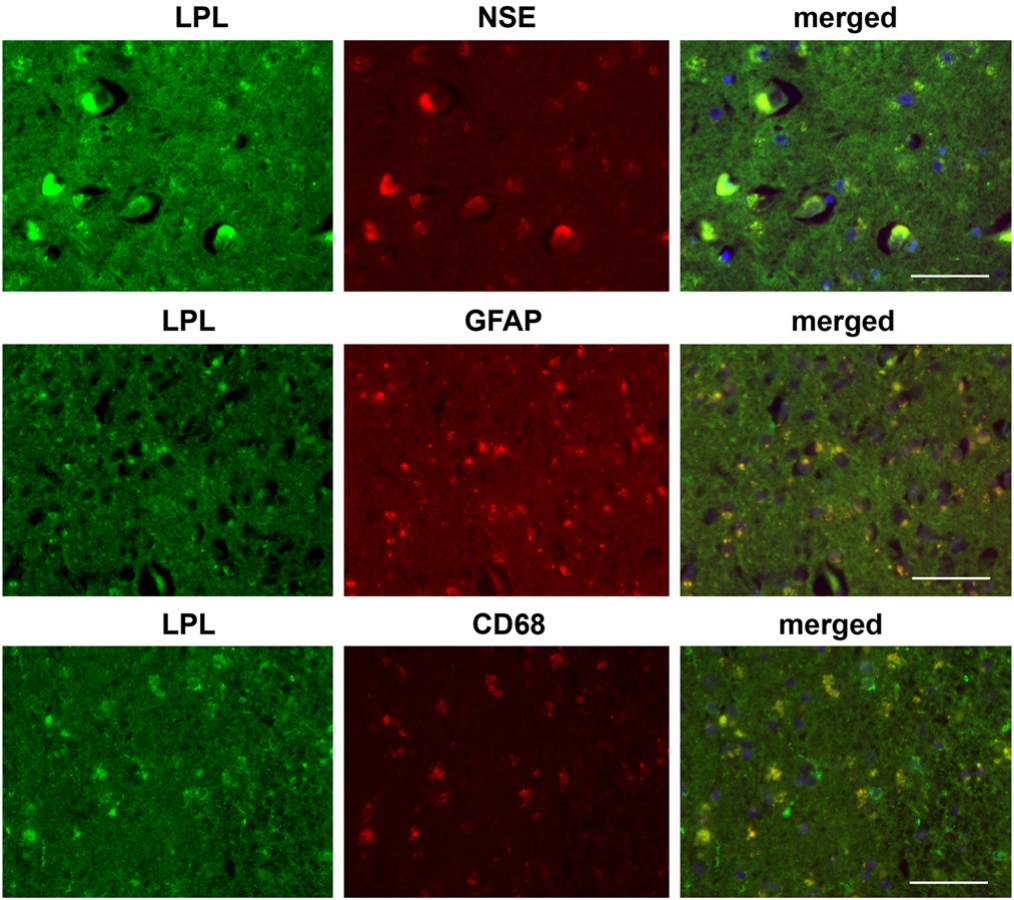
Colocalization with neuron-specific enolase (NSE; marker for neurons), glial acidic fibrillary protein (GFAP; marker for astrocytes) and CD68 (marker for microglia) in normal hippocampal brain tissue. Scale bar = 200 µm.

**Figure 3.**
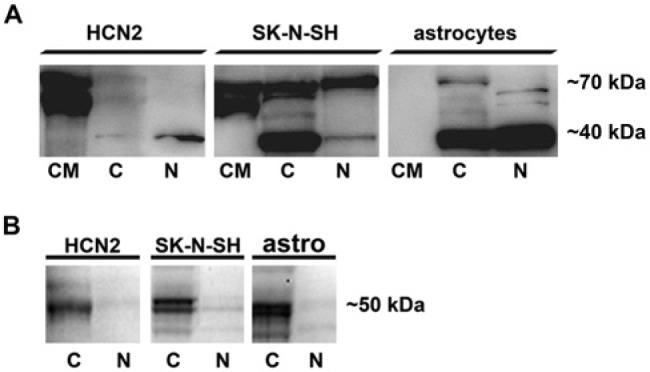
Lipoprotein lipase in conditioned media (CM), cytoplasmic (C) and nuclear (N) fractions of human cortical neurons (HCN2), human neuroblastoma (SK-N-SH) and primary human astrocytes, as determined by Western blotting. (A) LPL in HCN2, SK-N-SH cells and primary human astrocytes. (B) Beta-tubulin in C and N protein fractions of HCN2, SK-N-SH and primary human astrocytes; there was no significant contamination between the C and N protein fractions. Cells were incubated in DMEM under serum-free conditions at 37C, 5% CO_2_, 95% humidity for 24 h. Conditioned media were removed and centrifuged to remove cell debris, and cells were washed with PBS, scraped and the C and N protein fractions isolated using the NE-PER kit. Proteins were resolved by SDS-PAGE on a 4-12% Criterion XT gel, transferred to nitrocellulose membranes by semi-dry transfer, and incubated with mouse monoclonal antibody 5D2 against LPL or β-tubulin antibody overnight at 4C, followed by incubation with TrueBlot anti-mouse or anti-rabbit secondary antibody for 1 h at room temperature. The blot was developed using SuperSignal West Femto and documented by Kodak ImageStation CF440.

Colocalization studies using cellular markers confirmed the presence of LPL immunoreactivity in neurons, microglia and astrocytes (Fig. 2). Furthermore, LPL was secreted by neuronal cells *in vitro*, and was present in cytoplasmic and nuclear fractions of HCN2 and SK-N-SH cells (Fig. 3). In contrast, primary human astrocytes expressed LPL in cytoplasmic and nuclear fractions, but we did not detect LPL in the conditioned media obtained from cultured primary human astrocytes (Fig. 3).

**Figure 4.**
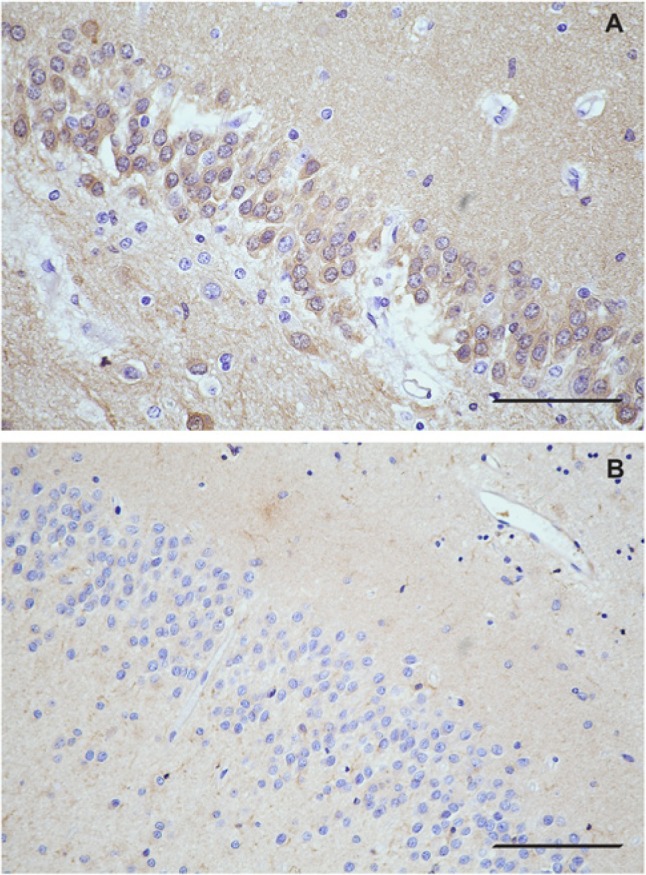
Dentate gyrus granule cells and the surrounding synaptic network are significantly less LPL immunoreactive in Alzheimer’s disease (AD) than in control hippocampal tissue. (A) Representative image of the dentate gyrus in healthy hippocampal tissue. (B) Representative image of the dentate gyrus area of hippocampus in AD. Note: all samples used for qualitative comparison of LPL distribution in healthy and AD brain were processed at the same time, and micrographs taken at the same time. Scale bar = 50 µm. A) is healthy; B) is AD.

In comparison with healthy subjects (Fig. 4A), AD patients had strikingly lower LPL immunoreactivity in the dentate gyrus (Fig. 4B). Granule cells of the dentate gyrus were poorly to moderately stained in AD samples, with comparatively enhanced synaptic network staining surrounding the dentate gyrus granule neurons; however, this staining was still weaker than that observed in healthy hippocampal tissue. In contrast, healthy dentate gyrus cells had strong cytoplasmic and nuclear staining immunoreactivity and robust staining of the dentate gyrus synaptic network (Fig. 4A). In healthy subjects, LPL immunoreactivity in this region was characterized by fine, well-defined, diffuse staining within the granule cells of the dentate gyrus and the surrounding synaptic network.

**Figure 5.**
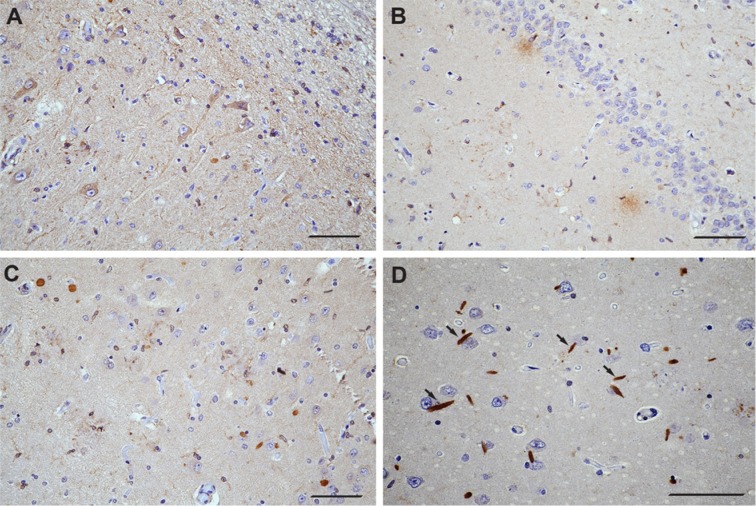
LPL immunoreactivity associated with different aspects of Alzheimer’s disease (AD) pathology in the hippocampal region. A) Low magnification micrograph of AD hippocampal region showing activated glial cells and diseased neurons. B) LPL immunostaining associated with diffuse plaques. C) LPL immunoreactivity in the areas of neurite pathology. D) The arrows point towards possible Hirano bodies. Scale bars = 50 µm.

In AD, LPL immunostaining was strongly associated with neurite pathology, including dystrophic and swollen axonal processes, diffuse neuritic plaques (Fig. 5), and some perivascular plaques. Possible Hirano bodies and activated glial cells stained as well (Fig. 5). LPL immunoreactivity that relates to neurite pathology was particularly strong in the CA1 neuronal layer. However, no LPL immunoreactivity was associated with neurofibrillary tangles or granulovacuolar degeneration. Strong immunoreactivity of the synaptic network was observed in the CA4 layer, consisting of both punctate and diffuse staining. The ependymal layer and choroid plexus showed strong LPL immunoreactivity, whereas arachnoid cap cells were relatively weakly immuno-positive, which was similar to the pattern of LPL staining of these structures in healthy brain tissues. No other consistent changes were observed in AD brain tissue compared with the samples obtained from healthy subjects.

**Figure 6.**
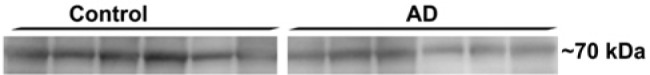
Lipoprotein lipase in human CSF samples obtained from control and Alzheimer’s disease (AD) subjects. Randomly selected CSF samples (10 µl) from control and AD subjects were resolved on 4-12% gel by SDS-PAGE, transferred to nitrocellulose membranes by semi-dry transfer and incubated with anti-LPL (5D2; 1:1000) and TrueBlot anti-mouse antibodies (1:2000). The blot was developed using SuperSignal West Femto and visualized with Kodak ImageStation CF400.

Analysis of CSF samples indicated that LPL levels are significantly reduced in CSF obtained from AD patients compared with healthy controls (relative intensity by densitometry scanning 3886 ± 1504 in control, *n*=27; 1980 ± 619 in AD, *n*=27; *p*<0.001; Fig. 6 shows a representative blot).

## Discussion

We evaluated the distribution of LPL in human brain tissue, and the differences in LPL distribution and levels in the brain tissue and CSF obtained from controls and AD patients. Previously published studies reported the presence of LPL mRNA, protein and activity in several regions of the mouse, rat, guinea pig and rabbit brain, with particularly high expression in hippocampal neurons, and relatively extensive distribution of LPL protein in rodent brain neurons (Goldberg et al. 1989; Yacoub et al. 1990; Tavangar et al. 1992; Vilaro et al. 1990; Paradis et al. 2004a; Wang and Eckel 2012). Brain LPL expression is reportedly regulated by nutritional and hormonal factors (Gavin et al. 1987), and its deficiency modifies energy balance and obesity *in vivo* (Wang H. et al. 2011). Published studies also indicate that LPL plays a role in cellular uptake of beta amyloid in astrocytes and neurons (Nishitsuji et al. 2011), which may be related to the bridging action of LPL (Eisenberg et al. 1992; Kanekiyo et al. 2011). Furthermore, LPL reportedly plays a role in neuronal differentiation (Paradis et al. 2003 and 2004b), and its levels are upregulated following ischemia-reperfusion injury *in vivo* (Wang et al. 2010).

In our study, LPL immunoreactivity was widely present throughout all major regions of human brain tissue, with moderate to high immunoreactivity in subgroups of neurons, extensive immunoreactivity in microglia, and sporadic immunoreactivity in astrocytes and oligodendroglia. LPL immunostaining in Schwann cells associated with cranial nerves is in agreement with previously published *in vitro* data (Huey et al. 1998 and 2002; Ferreira et al. 2002), and the presence of LPL in the brain microvasculature supports findings in the rat brain (Shirai et al. 1986).

Unexpectedly, we observed extensive nuclear staining in brain cells, including neurons. These findings are supported by analyses of neuronal cells *in vitro*, which indicate the presence of LPL in both cytoplasmic and nuclear compartments of neuronal cells and primary human astrocytes. Similarly, we detected LPL in cytoplasmic and nuclear fractions of cultured primary human astrocytes but, in contrast with neuronal cells, LPL was undetectable in the conditioned media of human astrocytes, indicating little, if any, LPL is secreted by astrocytes *in vitro*. As astrocyte immunoreactivity in the brain appeared more sporadic and relatively weak, except in reactive astrocytes, it is possible that LPL secretion from this cell type is tightly regulated, and possibly responsive to inflammatory stimuli.

Extensive LPL immunostaining in microglia throughout the brain tissue further support the notion that LPL may play an important role in the regulation of inflammatory processes in the brain, which may be similar to its functions in peripheral macrophages (Wållberg-Jonsson et al. 1996; Qiu et al. 2007; Jinno et al. 2011; Essaji et al. 2013; Takahashi et al. 2013). We observed much stronger LPL immunostaining in reactive glial cells, which was particularly striking in the hippocampal and temporal lobe samples obtained from patients with AD.

LPL immunoreactivity in the brain tissue obtained from patients with AD was marked by significant reduction of immunostaining in granule cells of the dentate gyrus and the surrounding synaptic network. The changes in the dentate cells are of particular interest because these cells have been shown to act as neuronal stem cells, and may be involved in neuronal regeneration (Altman and Das, 1965; Eriksson et al. 1998; Zhao et al. 2008; David et al. 2009; Deng et al. 2010; Winner et al. 2011; Hamilton et al. 2013). Fatty acids, particularly omega-3 fatty acids, play an important role in adult neurogenesis (Crupi et al. 2013). The physiological function of LPL in the regulation of fatty acid levels and availability may therefore be of importance to the process of neurogenesis. Reported findings that LPL levels are particularly high during embryonic brain development, and that LPL is important for neuronal differentiation and synaptic function support this notion (Yacoub et al. 1990; Tavangar et al. 1992; Nunez et al. 1995; Paradis et al. 2003 and 2004b; Xian et al. 2009; Wang and Eckel 2012). However, the potential role of LPL in adult neurogenesis is currently unknown. Previously published studies report that neurogenesis is significantly reduced in AD (Winner et al. 2011), and our findings that LPL immunoreactivity is strongly reduced in the granular layer of the dentate gyrus in AD hippocampal tissue may be connected with this observed deficit in neurogenesis in AD.

Studies of neuritic plaque material obtained from human brain indicated that LPL is present in amyloid plaques in AD (Rebeck et al. 1995). Our immunohistochemical analyses confirm these findings, showing significant LPL immunoreactivity in diffuse plaques. The presence of LPL in diffuse neuritic plaques, in reactive glia surrounding plaques, and in the vicinity of abnormal neurons suggest that upregulation of LPL in activated glial cells may be a compensatory response to neuronal pathology in AD.

Findings that LPL immunoreactivity is strongly present in areas that contain significant neuritic pathology (Shapiro and Eichenbaum 1999; Shao et al. 2011), such as CA1 dystrophic or swollen axonal processes, suggest that LPL functions may be relevant for the maintenance of axonal processes and connections. Our observations of the strong LPL staining of the synaptic network throughout the brain suggest that LPL has an important function at the level of the synapse. This notion is supported by *in vivo* studies, showing that LPL-deficient mice have a significantly reduced number of synaptic vesicles in presynaptic terminals and decreased levels of synaptophysin, which is associated with memory and learning insufficiency (Xian et al. 2009). Furthermore, LPL has been shown to be relevant for synaptic remodeling (Blain et al. 2004). Given the known LPL functions related to facilitating the uptake of lipoprotein-derived lipids (Eisenberg et al. 1992; Fagan et al. 1996; Medh et al. 1996; Mead et al. 2002; Merkel et al. 2002), its role in the transfer of α-tocopherol from periphery to the brain (Goti et al. 2002; Shi et al. 2010), and the relevance of these processes for synaptic function (Mauch et al. 2001; Göritz et al. 2005; Fester et al. 2009), it is plausible that LPL plays a pivotal role in creation, function and maintenance of the synaptic network. Furthermore, metabolism of DAG in the brain, which is likely affected by LPL, has been shown to modulate axonal guidance and synaptic plasticity (Oudin et al. 2011). Therefore, findings of significantly reduced levels of LPL in the CSF of AD patients, combined with a marked reduction of the synaptic network staining in AD dentate gyrus, may indicate that the observed decrease in LPL in AD could augment synaptic pathology in AD.

We also observed striking LPL immunoreactivity in rod-shaped perineuronal bodies in the CA1 layer that likely represent Hirano bodies. Although the presence of Hirano bodies has been reported in AD and in other neurodegenerative diseases (Perl 2010; Kaege et al. 2012), their function is still unclear (Myre 2012). Hirano bodies consist of actin filaments and actin-binding proteins, as well as proteins involved in activation of complement (Perl 2010; Satoh et al. 2013; Singhrao 2013). A recent study suggested that Hirano bodies reduce tau pathology *in vitro* (Furgerson et al. 2012). The functional significance of the strikingly elevated LPL immunoreactivity in the putative Hirano bodies is currently unclear, but may be a further sign of compensatory changes in LPL levels in areas that contain significant neurite pathology. In contrast to these findings, LPL immunoreactivity was noticeably absent from intraneuronal tangles, although another study previously reported significant association between LPL polymorphism and neurofibrillary tangle density in AD human brain (Blain et al. 2006); this suggests the possibility that LPL function in the brain indirectly affects formation of neurofibrillary tangles in AD.

In summary, LPL distribution in discrete populations of neurons, microglia, astrocytes and oligodendroglia throughout the brain suggest that LPL plays an important role in human brain. Strong, widespread association of LPL immunoreactivity with the synaptic network, in combination with previously published findings that LPL plays a role in the regulation of synapses, indicates the importance of LPL for formation and maintenance of healthy synapses. The observed changes in AD suggest that reduced levels of LPL in AD may significantly contribute to neurite pathology, and possibly contribute to the reduced neurogenesis potential observed in patients with AD.
